# Recurrence of positive SARS-CoV-2 viral RNA in recovered COVID-19 patients during medical isolation observation

**DOI:** 10.1038/s41598-020-68782-w

**Published:** 2020-07-17

**Authors:** Bo Yuan, Han-Qing Liu, Zheng-Rong Yang, Yong-Xin Chen, Zhi-Yong Liu, Kai Zhang, Cheng Wang, Wei-Xin Li, Ya-Wen An, Jian-Chun Wang, Shuo Song

**Affiliations:** 1 Science and Education Department, Shenzhen Samii Medical Center, 1 Jinniu West Road, Pingshan District, Shenzhen, Guangdong Province China; 2 HIV/AIDS Control and Prevention Division, Shenzhen Center for Disease Control and Prevention, Shenzhen, China

**Keywords:** Diseases, Health care, Medical research

## Abstract

Recently, the recurrence of positive SARS-CoV-2 viral RNA in recovered COVID-19 patients is receiving more attention. Herein we report a cohort study on the follow-up of 182 recovered patients under medical isolation observation. Twenty (10.99%) patients out of the 182 were detected to be SARS-CoV-2 RNA positive (re-positives), although none showed any clinical symptomatic recurrence, indicating that COVID-19 responds well to treatment. Patients aged under 18 years had higher re-positive rates than average, and none of the severely ill patients re-tested positive. There were no significant differences in sex between re-positives and non-re-positives. Notably, most of the re-positives turned negative in the following tests, and all of them carried antibodies against SARS-CoV-2. This indicates that they might not be infectious, although it is still important to perform regular SARS-CoV-2 RNA testing and follow-up for assessment of infectivity. The findings of this study provide information for improving the management of recovered patients, and for differentiating the follow-up of recovered patients with different risk levels.

## Introduction

The current pneumonia epidemic (COVID-19), caused by the SARS-CoV-2 coronavirus has spread to more than 200 countries. There have been more than 8 million confirmed cases and up to 440,000 deaths (as of June 18, 2020) ^1^, raising a high level of concern all over the world. Previous studies have mainly focused on the clinical and epidemiological characteristics of patients infected with SARS-CoV-2^2–4^. With the increase in the number of recovered patients, follow-up and detection are particularly important. Previous studies have found that patients who have recovered from COVID-19 are still testing positive for SARS-CoV-2^5–7^. A single center study reported that 7.41% of COVID-19 patients re-tested positive for SARS-CoV-2 RNA by real-time reverse transcriptase polymerase chain reaction (RT-PCR) test after discharge^8^, and this finding has challenged the current hospital discharge criteria for containing the pandemic. The present study analyzed the SARS-CoV-2 viral RNA test results in all 182 recovered COVID-19 patients in Shenzhen before April 21st during a 14-day medical isolation observation period, to provide more reference for containing the pandemic more effectively.

## Results

### Patients under 18 years old, and mild and moderately patients have a higher risk of re-testing positive

Among all the recovered and isolated patients, 182 of them satisfied the inclusion criteria of this study. They were all re-tested at least once. Eighty-four (46.2%) were males and 98 (53.8%) were females, and the average age was 46.4 ± 17.1 years (median 49 years, range 1–81 years). Thirty-nine (21.4%) had severe symptoms, and 143 (78.6%) had mild and moderate symptoms (Table 1). A few of them showed different symptoms (mild flu, allergic rhinitis, smoking-induced sore throat) during medical isolation, although COVID-19 symptoms did not recur.

**Table 1:** Basic information of recovered COVID-19 patients.

	Re-positive (n = 20)	Non-re-positive (n = 162)	*P* value
**Epidemiological information**			
Total (n = 182)	20 (10.99%)	162	/
Severe cases (n = 39)	0**	39	0.014
Wuhan exposure (n = 75)	5	70	0.120
Time from onset to admission	5.1 ± 4.8	4.5 ± 4.0	0.766
Time from admission to discharge	20.8 ± 7.1*	25.6 ± 7.6	0.02
**Comorbidity**			
Hypertension	3	26	0.907
Diabetes	0	12	0.211
Hyperlipemia	0	2	0.627
Cardiovascular disease	2	10	0.520
Malignant tumor	0	5	0.432
Hepatopathy	1	7	0.894
Lung disease	0	3	0.547
**Sex**			
Male (n = 84)	7 (8.3%)	77	0.294
Female (n = 98)	13 (13.3%)	85	
**Age (years)**			
Median age (range)	41.5 (1–72)	49 (1–81)	/
Average age	39.9 ± 20.1	47.2 ± 16.6	0.073
Under 18 years old (n = 13)	4 (30.8%)*	9	0.018
Over 18 years old (n = 169)	16 (9.5%)	153	

All data were analyzed using the Mann–Whitney U test. *p < 0.05, **p < 0.01 versus the non-re-positive group.

Twenty patients out of the 182 re-tested positive (13 females, seven males; 1–72 years old). Differences in sex, age, basic symptoms, and epidemiological information between those re-testing positive (re-positives) and those not re-testing positive (non-re-positives) were analyzed. The time from admission to discharge of the re-positives was significantly shorter than for the non-re-positives, indicating that the length of hospital stay might be important. There were no significant differences between re-positives and non-re-positives in terms of age median, sex, and comorbidities, although patients aged under 18 years had a higher re-positive rate (Table 1). Thirteen of them re-tested positive on the 7th day, and another 7 re-tested positive on the 14th day. Fourteen had positive nasopharyngeal swabs, and six had positive anal swabs. None had both swabs positive (Table 2).

**Table 2:** Recurrence of positive SARS-CoV-2 viral RNA in recovered COVID-19 patients.

Case number	Sex	Age (years)	Day 7 check		Day 14 check	
			Nasopharyngeal swab	Anal swab	Nasopharyngeal swab	Anal swab
Case 1	Male	38	Negative	Negative	Negative	Positive*
Case 2	Male	53	Negative	Negative	**Positive**	Negative
Case 3	Female	40	**Positive**	Negative	∕	∕
Case 4	Female	61	Negative	Negative	**Positive**	Negative
Case 5	Female	64	Negative	Negative	**Positive**	Negative
Case 6	Female	53	Negative	Negative	**Positive**	Negative
Case 7	Female	33	Positive*	Negative	∕	∕
Case 8	Female	1	Negative	**Positive**	∕	∕
Case 9	Female	34	Negative	Positive*	∕	∕
Case 10	Male	43	**Positive**	Negative	∕	∕
Case 11	Female	34	Negative	**Positive**	∕	∕
Case 12	Male	38	Negative	**Positive**	∕	∕
Case 13	Female	50	**Positive**	Negative	∕	∕
Case 14	Female	50	Positive*	Negative	∕	∕
Case 15	Female	5	Negative	**Positive**	∕	∕
Case 16	Female	55	**Positive**	Negative	∕	∕
Case 17	Female	72	Negative	Negative	**Positive**	Negative
Case 18	Male	54	Negative	Negative	Positive*	Negative
Case 19	Male	8	Negative	**Positive**	∕	∕
Case 20	Male	12	**Positive**	Negative	**/**	/

Bold indicates positive results.

*Results were weakly positive on the first test and Ct values were ≤ 40 when re-tested the next day.

/: Test was not performed.

**Figure 1:**
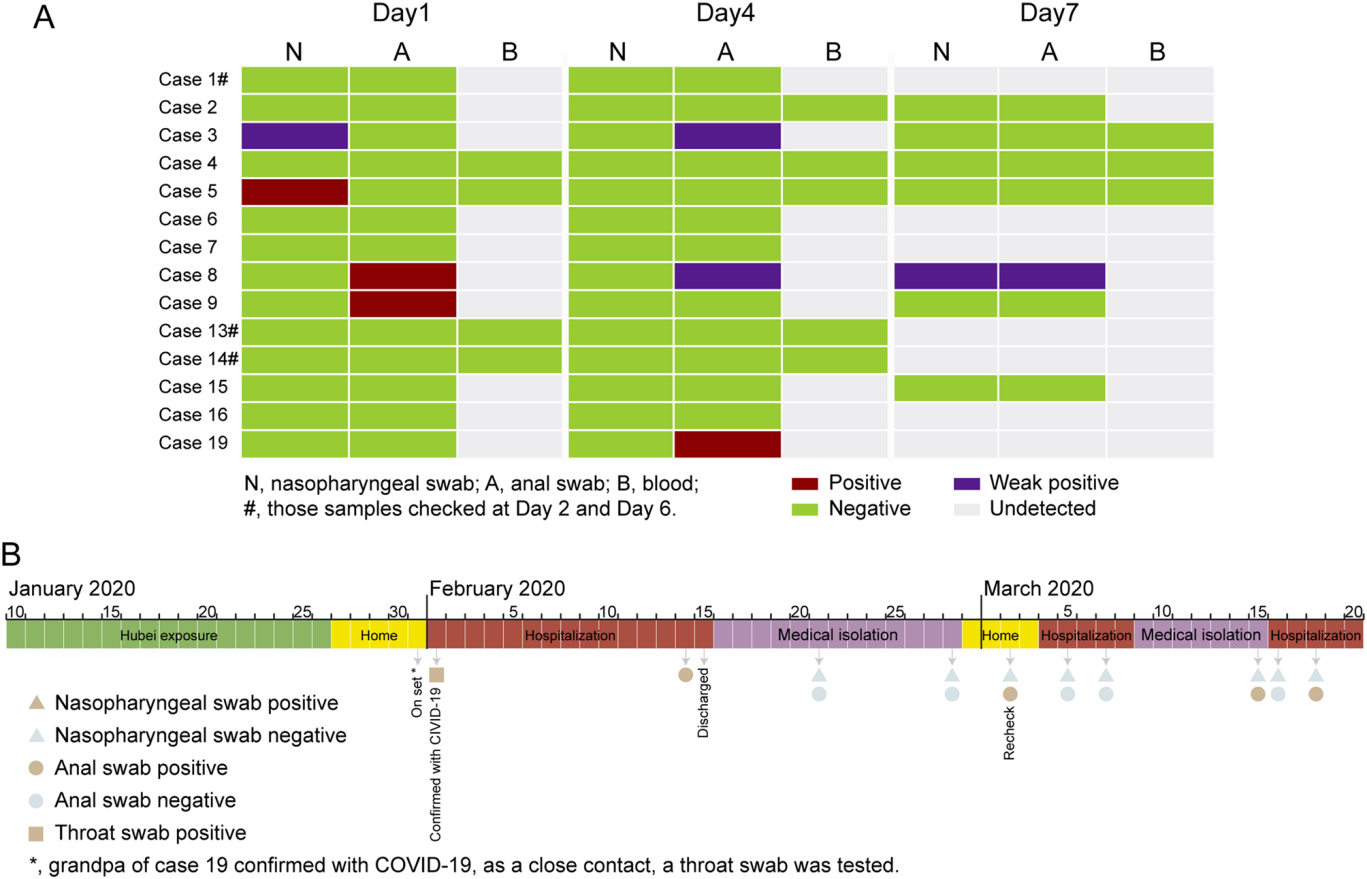
(**A**) RT-PCR testing of 15 re-positive cases out of 20. Data shows RT-PCR results of blood, nasopharyngeal swabs, and anal swabs tested on the 1st, 4th, and 7th day (2nd and 6th day for case 1, 13, and 14). (**B**) The timeline of case 19.

The re-positives were transferred to a designated hospital for quarantine treatment, and RT-PCR testing of blood, nasopharyngeal swabs, and anal swabs were on the 1st, 4th, and 7th day (some were taken on 2nd and 6th day). Among the results of the 14 cases, five were positive, and one of the five (case 8) was positive for tests on all three testing days. Three (cases 2, 4, and 15) of the 14 were negative for tests on all three testing days, and none have found positive results in blood tests (Fig. 1A).

### Re-positives and non-re-positives have the same level of antibodies

All the COVID-19 recovered patients were advised to undergo antibody detection and laboratory testing of blood. Fourteen out of the 20 re-positives, and 133 out of the 162 non-re-positives took the advice and underwent the tests. These tests revealed 13 negative results for IgA (13 non-re-positives and zero re-positives), one negative result for IgG (1 non-re-positive and zero re-positives), 42 negative results for IgM (38 non-re-positives and four re-positives), and positive total antibody (Ab) tests’ results for all 14 re-positives and 133 non-re-positives. Meanwhile, all 14 re-positives were SARS-CoV-2 antibody carriers. There were no significant differences between 133 non-re-positive recovered COVID-19 patients and 14 re-positives for total Ab, IgA, and IgG. The p-value for IgM was 0.024, but the median values were similar (2.66 and 3.16) (Figure S1). There were no obvious abnormalities found in routine laboratory blood testing (Table 3).

**Table 3:** Antibody detection and laboratory testing of SARS-CoV-2 viral RNA re-positive patients.

Case number	Ab(S/CO)#	IgA(S/CO)#	IgM(S/CO)#	IgG(S/CO)#	IL-6*	Hs-CRP*	SAA*	PCT*	Thyroid function				
									FT3*	FT4*	TSH*	TPO*	TG*
Case 1	138.57COI	3.07COI	**0.96COI**	18.10COI	61.65 pg/mL	0.03	< 5	0.065	2.81	15.35	1.68	13.90	14.40
Case 2	401.83COI	4.17COI	1.91COI	20.65COI	19.36 pg/mL	0.06	9.54	0.043	3.79	18.33	2.25	93.35	76.71
Case 3	343.34COI	1.33COI	2.23COI	20.83COI	< 1.50 pg/mL	0.12	< 5	0.01	2.46	12.12	2.98	33.55	313.20
Case 4	1,231.93COI	4.93COI	9.65COI	21.18COI	26.94 pg/mL	0.19	< 5	0.103	2.54	10.47	2.72	14.13	17.54
Case 5	19.56COI	5.33COI	**0.45COI**	8.09COI	14.00 pg/mL	0.10	5.28	0.098	2.76	13.86	3.26	15.20	256.20
Case 6	416.14COI	1.25COI	3.09COI	22.23COI	61.15 pg/mL	0.07	< 5	0.01	2.65	17.80	2.50	26.12	26.12
Case 7	1,224.68COI	5.52COI	6.32COI	19.87COI	< 1.50 pg/mL	0.06	8.47	0.01	3.34	25.37	1.10	21.37	24.90
Case 9	374.96COI	3.92COI	5.46COI	14.27COI	6.57 pg/mL	0.06	< 5	0.127	2.64	18.52	1.99	23.25	19.46
Case 11	73.36COI	1.18COI	**0.77COI**	16.79COI	< 1.50 pg/mL	0.07	< 5	0.01	2.87	19.53	1.19	7.47	11.94
Case 13	933.92COI	5.95COI	11.47COI	23.64COI	< 1.50 pg/mL	0.24	< 5	0.01	2.48	14.74	3.00	25.01	18.09
Case 14	559.62COI	7.11COI	3.39COI	23.68COI	< 1.50 pg/mL	0.03	< 5	0.01	2.34	15.15	1.82	16.07	15.06
Case 17	249.93COI	1.96COI	4.84COI	19.38COI	96.23 pg/mL	0.04	< 5	0.01	3.18	20.73	1.61	16.48	18.19
Case 18	222.17COI	5.83COI	3.65COI	14.76COI	108.84 pg/mL	0.11	< 5	0.113	4.21	14.74	0.91	67.45	63.39
Case 20	213.18COI	2.43COI	**0.33COI**	20.68COI	3.98 pg/mL	0.03	< 5	0.098	4.54	15.89	0.70	20.54	22.56

Bold indicates results that are S/COI < 1, which are negative results of IgM.

^#^S/CO < 1 was a negative result, S/CO ≥ 1 was a positive result for Ab, IgA, IgM, and IgG.

*Normal reference range: IL-6, < 7; Hs-CRP, 0–0.5; SAA, 0–10; PCT, 0–0.5; FT3, 2–4.4; FT4, 12–22; TSH, 0.27–4.2; TPO, 0–34; TG, 0–115.

### Asymptomatic carriers can be re-positive

We noticed the particular case of an 8-year-old boy (case 19) who had Hubei exposure history during Jan 10–16, 2020, who were re-tested positive for repeated times. He returned from a journey from Hubei to Shenzhen on Jan 26, 2020. His grandfather was confirmed to be infected with COVID-19 on Jan 31, 2020. Due to their close contact, throat swab tests were performed for the whole family and on Feb 1, 2020, the boy was confirmed as having COVID-19, and hospitalized. No fever or other symptoms were detected during his hospitalization. He reached the hospital discharge criteria (according to the 4th Trail edition) on Feb 15, but was requested to transfer to an isolation hotel for another 14 days (Feb 15–28) due to the positive results of an anal swab test on Feb 14. During hotel isolation, results on two test days (Feb 21 and 28) were negative, and thus he was allowed to go home. On his return hospital visit on Mar 2, an anal swab test showed a positive result, and he was hospitalized for a second time. During the second hospitalization, results on two test days (Mar 5 and 7) were negative, and he was transferred to isolation observation for a second period of 14 days from Mar 8. In the routine tests performed on Mar 15 (day seven of the second isolation observation), the anal swab test result revealed another positive result (Fig. 1B). Therefore he was hospitalized for a third time. During the third hospitalization, two further tests were performed, and there was a positive result (anal swab) on the 4th day at the designated hospital (Fig. 1A, case 19). It has been 35 days since his first recovery and discharge from hospital, and viral RNA remains, although there are no clinical symptoms.

## Discussion

None of the 182 discharged patients (under “Management of Diagnosis and Treatment of Novel Coronavirus Pneumonia Scheme Trial Version 7”) showed any COVID-19 related clinical symptomatic recurrence (fever, cough, respiratory tract disease, etc.) during the 14 days of medical isolation, indicating that COVID-19 responds well to treatment.

On the 7th and 14th day of discharge from hospital and transfer to isolation, we continued to discover positive results from nasopharyngeal swabs (7.69%) and anal swabs (3.3%) (n = 182). This suggested that recovered patients might still be carrying the virus. Discrepant results among re-positive recovered patients show that there are differences among individuals. Despite the positive results, positive results for either, as opposed to both, nasopharyngeal and anal swabs may suggest that these patients are recovering and there might be no replication of the virus within them. There have been no reports indicating that recovered patients are infectious, and a recent study of SARS-CoV-2 infected rhesus macaques shows that reinfection does not occur in recovered monkeys^9^. Thus, we believe that the positive results are evidence of the viral shedding process, and those patients are not infectious. Viral load is usually considered to be related to the outcome of the disease, but most re-positives show a normal range for inflammation markers (IL-6, CRP, SAA, PCT) (Table 3), suggesting that re-positive patients have no obvious disease progression or infectivity. At present, it is believed that RNA negative conversion generally takes 2–3 weeks. A recent study indicated that SARS-CoV-2 nucleic acid exists in feces for nearly 50 days^10^. The time from admission to discharge is shorter in viral RNA re-positive patients (Table 1), suggesting that the virus may not be completely eliminated due to the lighter symptoms and the faster attainment of the discharge standard. However, there is still reason for caution. A recent study found that SARS-CoV-2 viral particles remain in the lungs of patients in hospital whose nasopharyngeal swab test results are negative at three consecutive times^11^. This may explain, to some extent, why discharged patients retest positive. This is alarming and we need to pay more attention to recovered patients and their potential infectivity. We may need to reevaluate hospital discharge criteria, and the current patient management systems. Similar to previous studies’ findings^5,12^, most of our re-positive cases turned negative in the following tests, especially case 19. This case was an asymptomatic carrier, who re-tested positive twice, suggesting the need to further optimize the discharge criteria and medical isolation observation, which we are currently doing, after discharge from hospital.

Furthermore, patients under 18 years of age have much higher re-positive rates (30.8%) than the over 18-year-olds (9.5%). Most of the positive results (3/4) were found in anal swabs, and this indicates that juveniles have an increased risk of fecal–oral transmission^13,14^. In the present study, 39 (21.4%) of the 182 recovered patients in isolation were severely ill, although none of these re-tested positive. We assume that a stronger immune response is triggered in severely ill patients that restrains the virus more effectively. Previous studies found significant differences between the sexes in terms of morbidity and disease severity^2–4^, although for re-positives, there are no significant differences between the sexes, indicating that both sexes have similar shedding processes. A recent study showed that COVID-19 patients with any comorbidity yielded poorer clinical outcomes than those without^15^, but there are no such differences between re-positives and non-re-positives.

Zhao et al. reported that a higher titer of antibody in the plasma of patients with COVID-19 was independently associated with disease severity^16^. We analyzed the total Ab, IgM, IgG, and IgA, but there were no significant differences in antibody titers between re-positive recovered COVID-19 patients and non-re-positives (Figure S1), suggesting that all the 182 recovered patients, including the 20 cases that re-tested positive, are antibody carriers. Furthermore, we did not find an association between viral load (Table S1) and antibody titer (Table 2). This may suggest that the re-positives are shedding viral RNA segments, and re-testing positive does not cause an inflammatory response or antibody level fluctuations.

Taken together, patients aged under 18 years, and mild and moderately ill patients have a higher risk of recurrence of positive SARS-CoV-2 viral RNA via an RT-PCR test, although all ages and sexes are at risk of re-testing positive. No severely ill patients were re-tested positive in our study, although these results are not sufficient to prove that severely patients are not at risk from re-testing positive. All discharged patients should undergo medical observation and quarantine for at least 14 days. Longer periods of observation and surveillance might be necessary.

## Methods

As of Feb 21, 2020, COVID-19 patients of Shenzhen city who met all of the hospital discharge criteria were requested to stay in medical isolation observation for a further 14 days, and the discharge criteria includes:

1. Body temperature below 37 degrees, lasting for at least three consecutive days;
2. Resolved respiratory symptoms;
3. Substantially improved chest lesions on computed tomography (CT) images; and
4. Two consecutive negative RT-PCR test results with at least a 1-day interval^17^.

The clinical classification of COVID-19 is defined clearly in the “Diagnosis and Treatment of Pneumonia Caused by Novel Coronavirus (Trial Version 7)”. In brief, the mild type has no signs of pneumonia on chest imaging; the moderate type includes fever and respiratory symptoms, and signs of pneumonia on radiologic assessment; the severe type meets any of the following criteria: (1) shortness of breath, RR ≥ 30 times/min; (2) oxygen saturation ≤ 93% at rest; (3) arterial oxygen partial pressure/fraction of inspiration O_2_ (PaO_2_/FiO_2_ ≤ 300 mmHg); and (4) pulmonary imaging showing significant progression of lesion > 50% within 24–48 h^17^.

### Cohort information

Patients infected with COVID-19 were divided into severe and non-severe (mild and moderate) groups according to the guidelines for “Diagnosis and Treatment of Pneumonia Caused by Novel Coronavirus (Trial Version 7)” ^17^. All the discharged patients were asked to stay in medical isolation observation for a further 14 days at the Samii Medical Center, in a single room for each patient. Viral RNA testing of nasopharyngeal swabs and anal swabs were carried out on the 7th and 14th day. Antibody detection and laboratory testing of blood were carried on the 7th day.

### RT-PCR analysis

Nasopharyngeal swabs and anal swabs were taken on the 7th and 14th day of observation, for RT-PCR tests at the Shenzhen Center for Disease Control and Prevention (CDC), to decide if they were allowed to go home. The RT-PCR tests were performed by the CDC using the High Pure Viral RNA Kit (Roche, Mannheim, Germany) and the 2019-nCoV Viral RNA detection kit (Bio-Germ, Shanghai, China), a similar methods have been described previously^18^. In brief, we put nasopharyngeal swabs/anal swabs into a collection tube with 1.5 mL of virus preservation solution. Then 200 μL of cell lysate was vortexed for 10 s and was then allowed to stand at room temperature for 10 min. We then collected the suspension after a 10-min centrifugation at 1,000 rpm. Two target genes of SARS-CoV-2, including the open reading frame 1ab (ORF1ab) and the nucleocapsid protein (N), were simultaneously amplified and tested during the RT-PCR assay. Target 1 (ORF1ab): forward primer CCCTGTGGGTTTTACACTTAA; reverse primer ACGATTGTGCATCAGCTGA; probe: 5′-VIC-CCGTCTGCGGTATGTGGAAAGGTTATGG-BHQ1-3′. Target 2 (N): forward primer GGGGAACTTCTCCTGCTAGAAT; reverse primer CAGACATTTTGCTCTCAAGCTG; probe 5′-FAM- TTGCTGCTGCTTGACAGATT-TAMRA-3′. A cycle threshold value (Ct-value) less than 37 was defined as positive, and a Ct-value no less than 40 was defined as negative. A medium load, 37 ≤ Ct < 40, was defined as weakly positive, which requires further confirmation by re-testing. If Ct-value ≤ 40 in the re-test on the next day, a positive result would be reported.

We collected all of the RT-PCR test information from the recovered and isolated for 7 + days COVID-19 patients, and analyzed the re-positive tests results.

### Antibody detection and laboratory testing

The main results and indicators of epidemiology, demography, clinical manifestation, and laboratory examinations of 182 recovered patients with COVID-19 were collected and analyzed. The Inflammation markers and thyroid functions were tested, including Interleukin-6 (IL-6), hypersensitive-c-reactive-protein (Hs-CRP), serum amyloid A protein (SAA), procalcitonin (PCT), serum free triiodothyronine (FT3), free tetraiodothyronine (FT4), thyroid stimulating hormone (TSH), thyroid peroxidase (TPO), and thyroglobulin (TG). Total Ab, IgA, IgG and IgM were tested on the 7th day using a SARS-CoV-2 testing kit (WANTAI BioPharm, Beijing, China) based on the Chemiluminescence method. All tests were performed according to the manufacturer’s instructions. S/CO < 1 indicated a negative antibody result, and S/CO ≥ 1 indicated a positive antibody result.

This study was approved by the Shenzhen Samii Medical Center Institutional Review Board (SSMC-R-20200401) and we declare that all the patients involved in this study have been fully informed and written informed consents were obtained. Concerning the 20 minors involved in the study, their patients/LARs have been informed and signed the informed consents on their behalf. These data do not contain any private information of the patients. All methods were performed in accordance with the relevant guidelines and regulations.

### Statistical analysis

The Mann–Whitney U test was used to analyze differences in basic information between non-re-positive recovered COVID-19 patients and re-positives. A two-tailed independent sample t-test was used to test for significant differences in antibody detection between non-re-positives and re-positives. The Mann–Whitney U tests were performed using ggplot2, and the two-tailed independent sample t-tests were performed using the ggpubr package of R software (version 3.6), respectively.

## Supplementary information

Supplementary information

## Data Availability

The datasets used and/or analyzed during this study are available from the corresponding author upon reasonable request.
