# Supplementary material for: Recurrence of positive SARS-CoV-2 viral RNA in recovered COVID-19 patients during medical isolation observation
Source: Sci Rep. 2020 Jul 17;10:11887. doi: 10.1038/s41598-020-68782-w (PMC7368008; doi:10.1038/s41598-020-68782-w)
Supplement: Supplementary file 1 — Supplementary information [file 41598_2020_68782_MOESM1_ESM.pdf]

Recurrence of Positive SARS-CoV-2 Viral RNA in Recovered COVID-19 Patients During Medical Isolation Observation

Recurrence of Positive SARS-CoV-2 Viral RNA in Recovered COVID-19 Patients During Medical Isolation Observation

Bo Yuan1#, Han-Qing Liu1#, Zheng-Rong Yang2, Yong-Xin Chen1, Zhi-Yong Liu1, Kai Zhang1, Cheng Wang1, Wei-Xin Li1, Ya-Wen An1, Jian-Chun Wang1*, Shuo Song1*

Author affiliations: Science and Education department, Shenzhen Samii Medical Center（B. Yuan, H. Liu, J. Wang, Y. Chen, Y. An, C. Wang, W. Li, S. Song）; HIV/AIDS Control and Prevention Division, Shenzhen Center for Disease Control and Prevention (Z. Yang)

# These authors contributed equally to this work.

* Correspondence to Shuo Song and Jian-Chun Wang, 1 Jinniu West Road, Pingshan district, Shenzhen, Guangdong province, China. Email: songshuo@ssmc-sz.com; wangjianchun@ssmc-sz.com.

Figure S1. The comparison of antibody levels against SARS-CoV-2 among 133 none re-positive recovered COVID-19 patients and 14 re-positives. The average S/CO value of total Ab, IgA, IgG and IgM were compared between re-positives (green) and none re-positives (red). Black dot shown a positive result (S/CO ≥ 1), and red dot shown a negative result (S/CO < 1). A two tailed independent sample t-test was used to show the significant difference between the two groups.

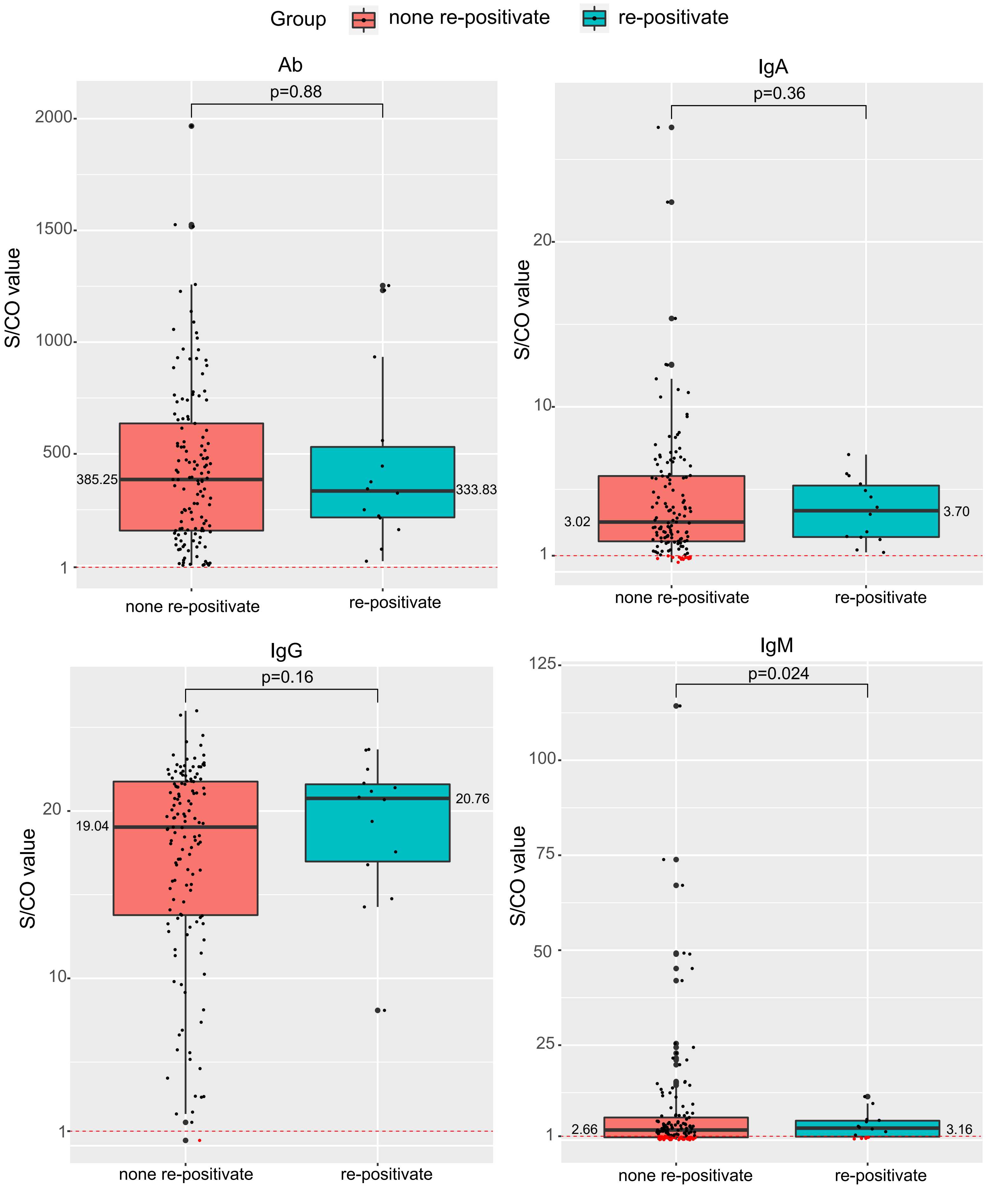
Group
none re-positivate
re-positivate
Ab
p=0.88
2000
1500
1000
500
1
385.25
333.83
S/CO value
none re-positivate
re-positivate
IgA
p=0.36
20
10
1
3.02
3.70
S/CO value
none re-positivate
re-positivate
IgG
p=0.16
20
10
1
19.04
20.76
S/CO value
none re-positivate
re-positivate
IgM
p=0.024
125
100
75
50
25
1
2.66
3.16
S/CO value
none re-positivate
re-positivate

Table S1 Ct Value of 19 re-positive cases of SARS-CoV-2 Viral RNA RT-PCR test

		Ct value		Ct value (re-test)	
	swab	FAM(N)	VIC(ORF1ab)	FAM(N)	VIC(ORF1ab)
Case 1*	anal swab	-	40	33.18	38.25
Case 2	nasopharyngeal swab	31.19	34.75		
Case 4	nasopharyngeal swab	32.79	36.14		
Case 5	nasopharyngeal swab	32.98	37.83		
Case 6	nasopharyngeal swab	32.35	36.74		
Case 7*	nasopharyngeal swab	**37.5**	**40**	37.07	39.01
Case 8	anal swab	33.22	37.14		
Case 9*	anal swab	38.5	39.5	39	40
Case 10	nasopharyngeal swab	27.87	31.5		
Case 11	nasopharyngeal swab	32.9	36.43		
Case 12	nasopharyngeal swab	32.26	36.43		
Case 13	nasopharyngeal swab	37	39.5		
Case 14*	nasopharyngeal swab	**39**	**-**	34.69	39.12
Case 15	anal swab	35.13	-		
Case 16	nasopharyngeal swab	36.5	39		
Case 17	nasopharyngeal swab	35.5	38		
Case 18*	nasopharyngeal swab	**37.92**	**-**	39	-
Case 19	anal swab	33	35		
Case 20	nasopharyngeal swab	37.5	38.5		

-: $\triangle$Ct > 40, no Ct value shown.

*: Ct values are weak positive (37 < $\triangle$Ct ≤ 40) for both N protein and ORF1ab, RT-PCR re-test was done next day, if Ct value ≤ 40, a positive result would be reported.

Here we only get Ct value of 19 re-positive cases, the Ct value of Case 3 was missed.
